# Supplementary material for: Enhancer decommissioning by MLL4 ablation elicits dsRNA-interferon signaling and GSDMD-mediated pyroptosis to potentiate anti-tumor immunity
Source: Nat Commun. 2022 Nov 2;13:6578. doi: 10.1038/s41467-022-34253-1 (PMC9630274; doi:10.1038/s41467-022-34253-1)
Supplement: Supplementary file 1 — Supplementary Information [file 41467_2022_34253_MOESM1_ESM.pdf]

Supplementary information for

Enhancer decommissioning by MLL4 ablation elicits dsRNA-interferon signaling and GSDMD-mediated pyroptosis to potentiate anti-tumor immunity

Hanhan Ning[1#], Shan Huang[1#], Yang Lei[3#], Renyong Zhi[1], Han Yan[1], Jiaxing Jin[1], Zhenyu Hu[3], Kaimin Guo[3], Jinhua Liu[1], Jie Yang[4], Zhe Liu[5], Yi Ba[2], Xin Gao[3,*], Deqing Hu[1,2*]

Correspondence to: Deqing Hu (hudq@tmu.edu.cn) or Xin Gao (gaoxin1@ihcams.ac.cn)

This PDF file includes:

Supplementary figures 1 to 8

Supplementary Figure 1

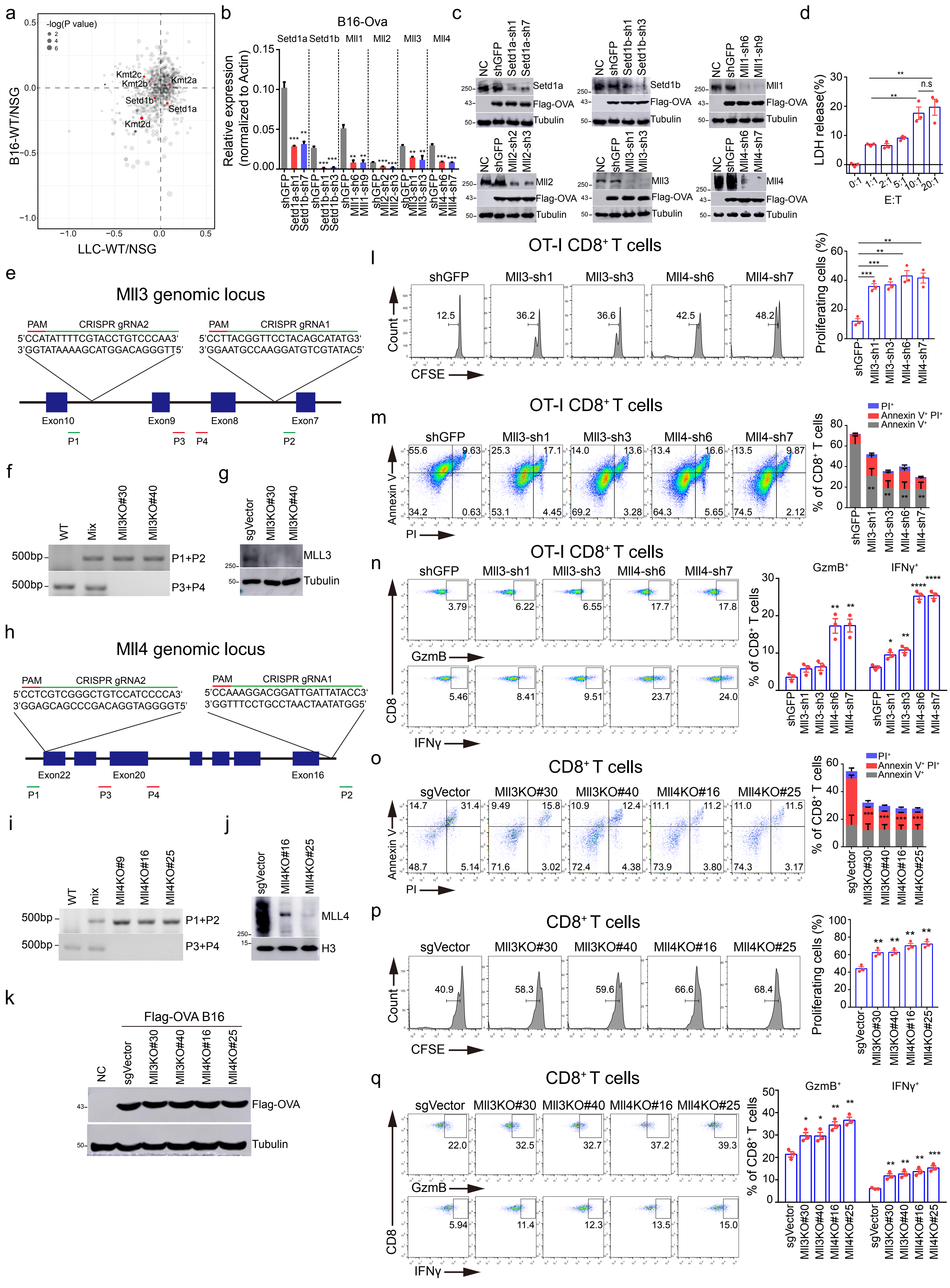

Supplementary figure legends

Supplementary Figure 1. Tumor-cell intrinsic MLL3 and MLL4 restrain the cytotoxic activity of CD8$^+$ T cells *in vitro*, related to Figure 1.

(a). Scatter plots showing *in vivo* CRISPR/Cas9 screen for tumor cell-intrinsic factors regulating host immune response to B16 and LLC- derived tumors. Datasets are from Gabriel K Griffin, et al[1] and re-analyzed in this work.

(b). Knocking down efficiency of indicated H3K4 methyltransferase was determined by RT-qPCR (mean ± SD, n=3).

(c). Expression of indicated H3K4 methyltransferase and Flag-tagged ovalbumin (Ova) was measured by immunoblotting. Shown are representative results from two biological replicates

(d) Titrating the optimal ratio of OT-I CD8$^+$ T to B16-Ova (effector to target, E/T) for tumor cell killing as measured by LDH release.

(e and h). Strategies for *Mll3* (e) and *Mll4* deletion (h) by CRISPR/Cas9. Genotyping primers are represented as color-coded bars.

(f and i). PCR genotyping results for two independent clones of *Mll3* (f) or *Mll4* (i) knockout B16-Ova cells.

(g, j, and k). Immunoblotting analyses of MLL3 (g), MLL4 (j) or OVA (k) level in two independent *Mll3* or *Mll4* knockout B16-Ova clones. Shown are representative results from two biological replicates**.**

(l-n). OT-I CD8$^+$ T cells were incubated with B16-Ova cells with indicated knockdown at an E/T ratio of 10:1 for 24 hours. Proliferation (l), viability (m), and cytotoxicity (n) of OT-I T cells were determined by flow cytometry analyses.

(o-q). Indicated B16-Ova cells were co-cultured with naïve CD8$^+$ T cells at an E/T ratio of 10:1 in the presence of anti-CD3 and -CD28 antibodies for 24 hours. Cell death and the proliferative and cytotoxic capacity of CD8$^+$ T cells were analyzed by flow cytometry.

Quantification in (d, l to q) was from three biological replicates and shown as mean±SEM, with statistical significance determined by two-tailed unpaired t test. P values were computed by two-sided unpaired t test in (a) **P < 0.01, ***P < 0.001

Supplementary Figure 2

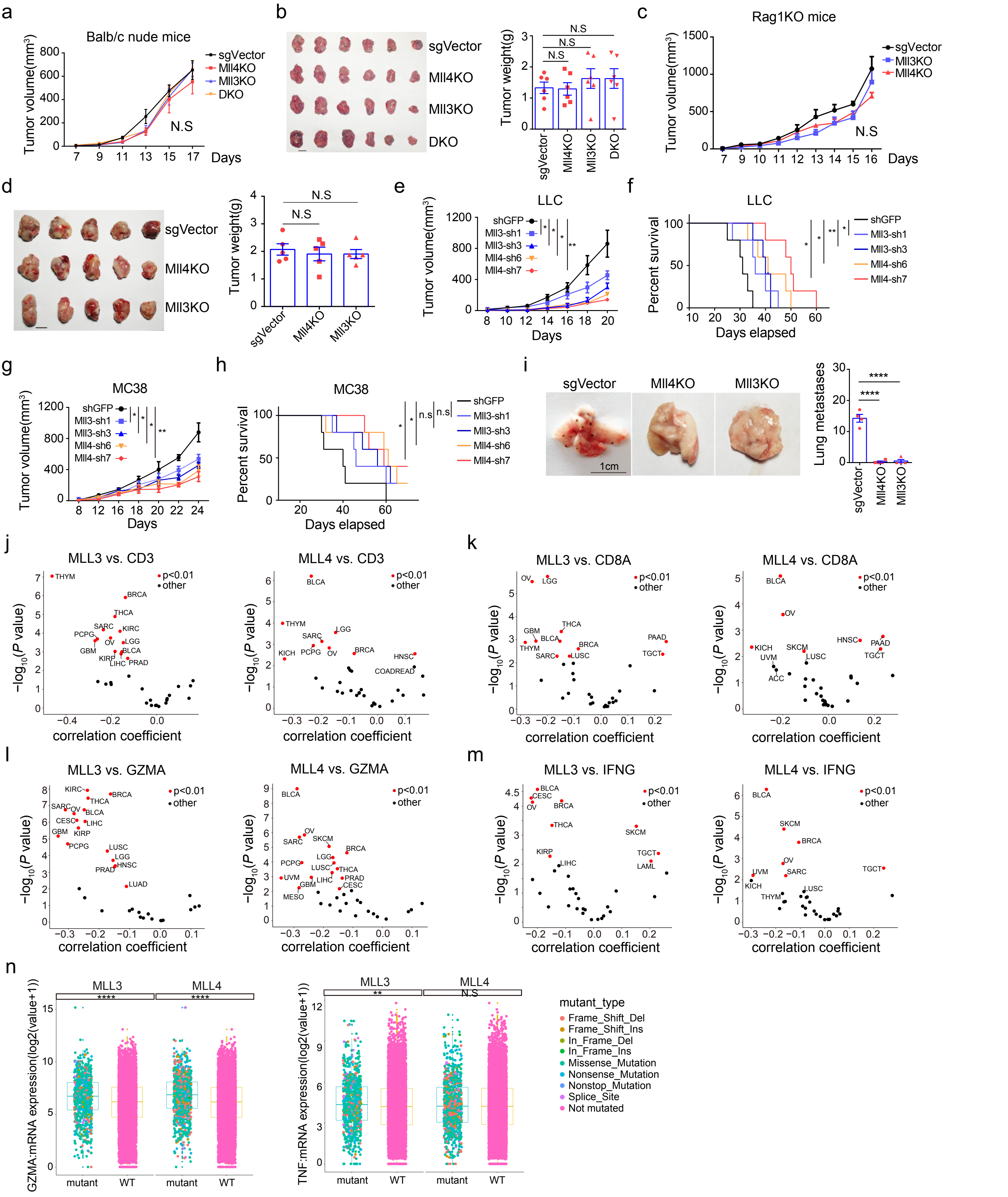

Supplementary Figure 2. Inactivation of *Mll3* or *Mll4* in tumor cells promotes anti-tumor immunity of $CD8^+$ T cells to attenuate tumor progression *in vivo*, related to Figure 2.

(a and b). Tumor growth over time (a) and tumor images and weight quantification at the time of experimental endpoint in BALB/c nude mice subcutaneously implanted with indicated B16 cells (b) (mean ±SEM, n=6).

(c and d). Tumor growth over time (c) and tumor photographs and weight quantification of indicated tumors at the time of experimental endpoint in *Rag1*$^{-/-}$ mice (d) (mean ±SEM, n=5).

(e and f). LLC cells lentivirally transduced with indicated shRNAs were inoculated subcutaneously into C57BL/6J mice. Tumor growth was monitored every 2 days from day 8 to 20 post implantation (e). Kaplan-Meier survival curves for mice are shown in (f) (mean ±SEM, n=5).

(g and h). MC38 cells lentivirally transduced with indicated shRNAs were inoculated subcutaneously into C57BL/6J mice. Monitoring of tumor growth was conducted every 2 days from day 8 to 24 post implantation (g). Kaplan-Meier survival curves for mice are shown in (h) (mean ±SEM, n=5).

(i). Lung images and quantification for colonization of control, *Mll3*$^{-/-}$ or *Mll4*$^{-/-}$ B16 cells 14 days post intravenous injection into C57BL/6J mice (mean ±SEM, n=4).

(j-m). Volcano plots showing the Spearman's correlation and estimated significance for the expression of *MLL3* or *MLL4* with mRNA levels of *CD3* (j), *CD8A* (k), *GZMA* (l), and *IFNG* (m) in RNA-seq datasets across TCGA cancer types[2]. Each dot represents a cancer type and significant correlations are highlighted in red.

(n). Boxplots showing expression of cytotoxic effectors in TCGA human pan-cancer samples that are stratified to harbor wild-type or genetically altered in *MLL3* or *MLL4*. Center line, median; box bounds, upper and bottom quantiles; whisker, 1.5 × IQR.

Statistical significance was determined by two-tailed unpaired t (b, d and i), two-way ANOVA (a, c, e and g), log-rank (Mantel–Cox) test (f and h), cor.test (j-m) or Wilcoxon test (n). *P < 0.05,**P < 0.01, ***P < 0.001.

Supplementary Figure 3

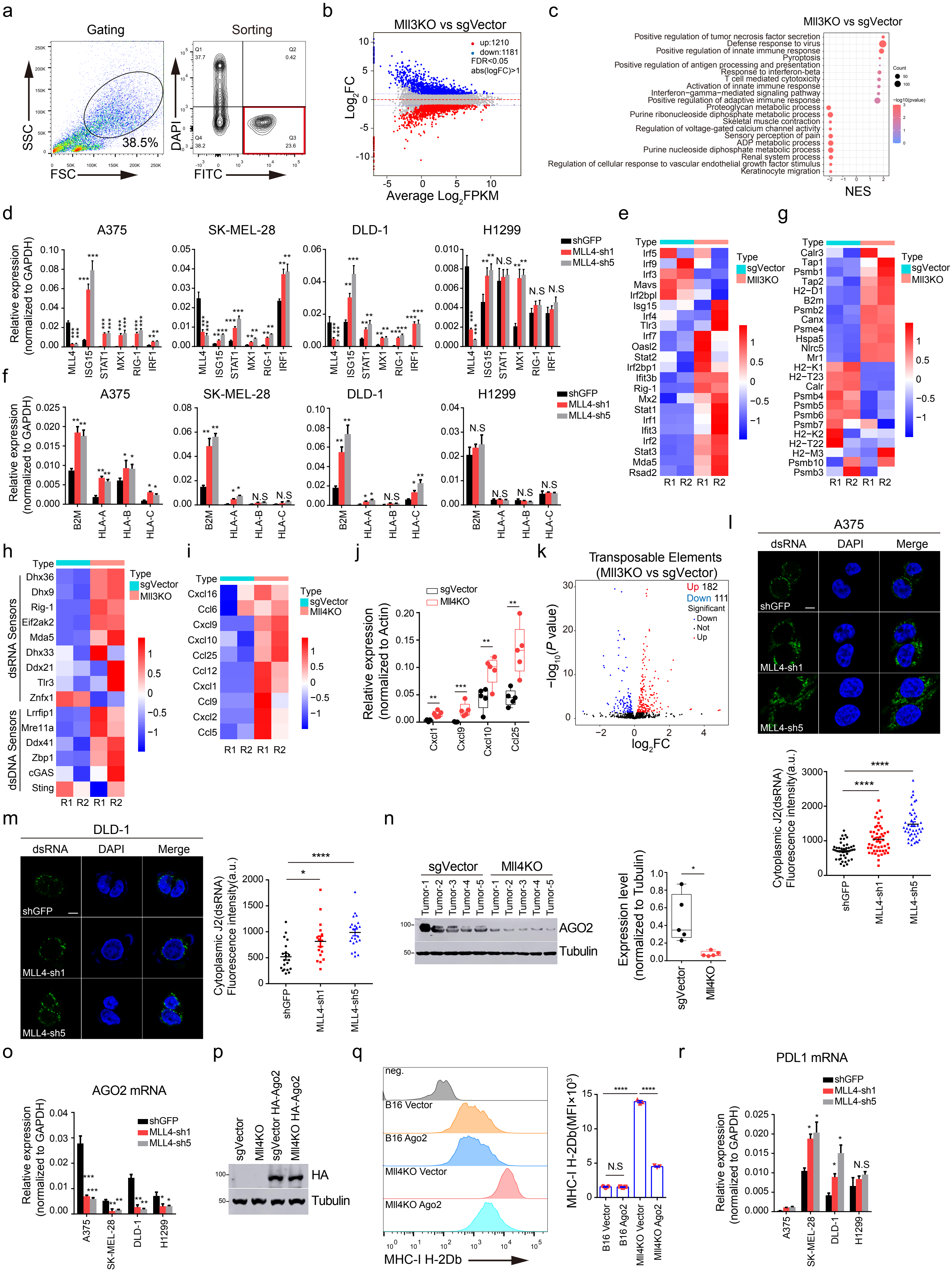

Supplementary Figure 3. Loss of *Mll3* and *Mll4* elicits interferon signaling and anti-tumor immune response, related to Figure 3.

(a). Gating strategies for isolating GFP-labeled B16 tumor cells from C57BL/6J by FACS.

(b and k). MA and volcano plots showing differentially expressed protein-coding and ERV transcripts in *Mll3*$^{-/-}$ B16 tumor cells of C57BL/6J mice (n=2).

(c). GSEA of the differentially expressed genes in *Mll3*$^{-/-}$ B16 tumor cells of C57BL/6J mice (n=2).

(d and f). mRNA levels of indicated genes in human cancer cell lines were measured by RT-qPCR and expressed as mean ± SD from three technical triplicates in one of biological replicates.

(e, g, h and i) Heatmaps for the expression of interferon-responsive genes (e), MHC I machinery (g), dsRNAs and dsDNAs sensors (h), and chemokines (i) in indicated B16 tumor cells of C57BL/6J mice (n=2).

(j and n). Levels of representative chemokine mRNAs and AGO2 protein in control and *Mll4*$^{-/-}$ B16 bulk tumors of C57BL/6J mice were analyzed by RT-qPCR and immunoblotting, respectively, and shown in box plot. Center line, mean; box bounds, upper and lower quartile; whisker, maximal and minimal data points (n=5).

(l and m) Representative images of intracellular dsRNA immunostaining in A375 and DLD-1 cells with *MLL4* depletion. Quantifications of dsRNA fluorescent intensity were shown as mean±SEM from more than 40 and 15 cells in A375 and DLD-1 cells, respectively. Scale bar, 10 μm.

(o and r). mRNA levels of *AGO2* and *PD-L1* in indicated human cancer cell lines with MLL4 depletion was measured by RT-qPCR and shown as mean±SD from technical triplicates in one of biological replicates.

(p). Immunoblotting analysis of ectopically expressed HA-tagged *Ago2* in control and *Mll4*$^{-/-}$ B16 cells. Shown are representative results from one representative of two biological replicates

(q). Cell surface level of free MHC I in indicated B16 cells were shown as mean±SEM. from three biological replicates.

Statistical significance was determined by quasi-likelihood F test (b and k), permutation test (c), or two-tailed unpaired t (d, f, j, l, m, n, o, q and r). *P < 0.05,**P < 0.01, ***P < 0.001.

Supplementary Figure 4

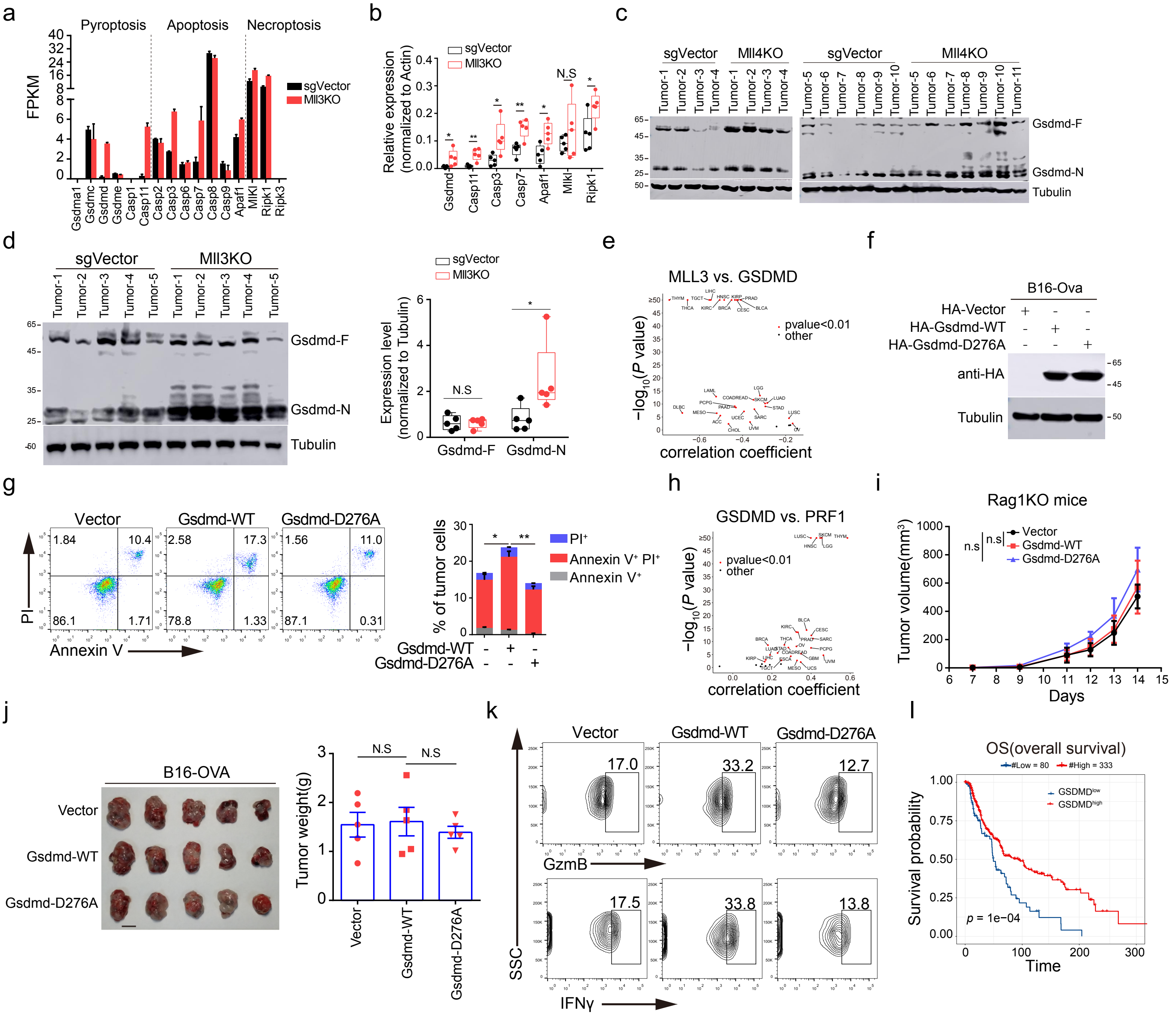

Supplementary Figure 4. Tumor cell ablation of *Mll3* and *Mll4* elevates the expression of genes responsible for GSDMD-mediated pyroptosis, related Figure 4.

(a). Histograms showing expression of in indicated genes in sorted control and *Mll3*$^{-/-}$ B16 tumor cells (mean±SEM, n=2).

(b and d). mRNA levels of indicated genes and GSDMD protein abundance in control and *Mll3*$^{-/-}$ B16 bulk tumors was analyzed by RT-qPCR and immunoblotting, respectively, and shown in box plot. Center line, mean; box bounds, upper and lower quartile; whisker maximal and minimal data points (n=5).

(c). Immunoblotting analyses of GSDMD expression in control and *Mll4*$^{-/-}$ B16 bulk tumors (sgVector, n=10; Mll4KO, n=11). Representative results are shown from two biological replicates.

(e and h). Volcano plots showing TCGA pan-cancer RNA-seq analyses of the Spearman's correlation for indicated pairs. Each dot represents a cancer type and significant correlations are highlighted in red.

(f). Immunoblotting of cellular extracts of B16-Ova cells 36 hours post lentiviral transduction of indicated construct.

(g). Representative flow cytometry scatter plots showing cell death of vehicle, N-terminally HA-tagged WT, or D276A mutant *Gsdmd*-reconstituted B16-Ova cells co-cultured with OT-I CD8^{+} T cells at an E/T ratio of 10:1 for 24 hours. Quantification was shown as mean±SEM (n=3) .

(i and j). *Rag1*$^{-/-}$ mice (n=5) were implanted with B16-Ova cells expressing vehicle, N-terminally HA-tagged WT, or D276A mutant *Gsdmd*. Tumor growth over time (i) and tumor images and their weight quantification at the time of mice sacrifice were shown as mean ±SEM (j).

(k). Flow cytometry analyses of the intratumoral CD8^{+} T cell cytotoxicity as determined by the levels of IFNγ and GzmB.

(l). Kaplan-Meier plots comparing the overall survival of TCGA SKCM patients grouped according to the expression level of GSDMD (GSDMD^{low}, n=80; GSDMD^{high}, n=333)

Statistical significance was determined by two-tailed unpaired t (b, d, g, h, and j), cor.test (e and h), two-way ANOVA test (i) and log-rank test (l). *P < 0.05,**P < 0.01, ***P < 0.001.

Supplementary Figure 5

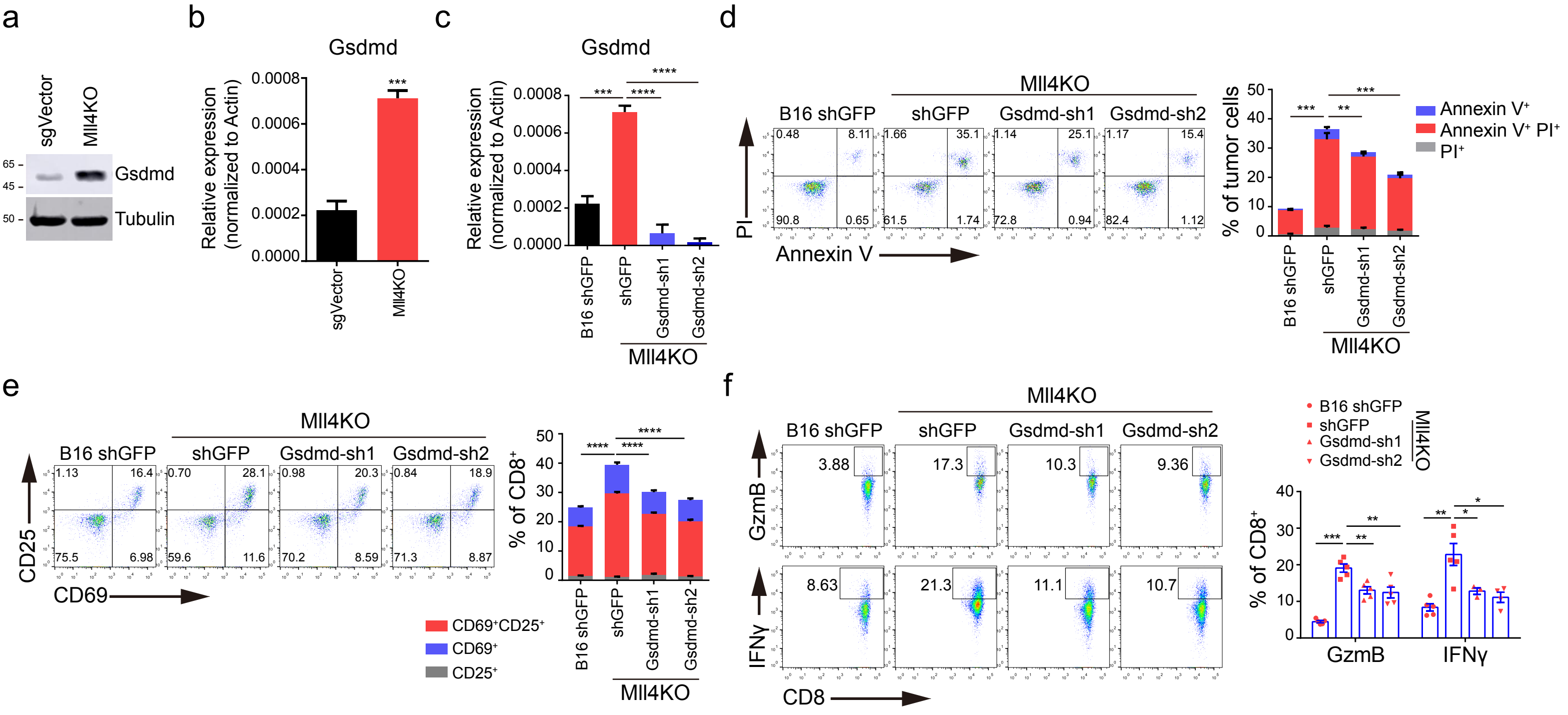

Supplementary Figure 5. Upregulation of GSDMD promotes activation and cytotoxic capacity of CD8^{+}T cells *in vitro*, related to Figure 5.

(a and b). Immunoblotting (a) and RT-qPCR (b) analyses of *Gsdmd* expression in control and *Mll4*$^{-/-}$ B16-Ova cells. Expression was shown as mean ±SD from technical triplicates in one experiment of two biological replicates.

(c). RT-qPCR analysis of *Gsdmd* expression in control or *Mll4*$^{-/-}$ B16-Ova cells lentivirally transduced with shRNAs targeting *Gsdmd*. Expression was shown as mean ±SD from technical triplicates in one experiment of two biological replicates.

(d). Control or *Mll4*$^{-/-}$ B16-Ova cells lentivirally transduced with shRNAs targeting *Gsdmd* were incubated with OT-1 CD8^{+} T cells at an E/T ratio of 10:1 for 24 hours. Cell death was analyzed by flow cytometry and shown as mean ±SEM from four biological replicates.

(e and f). Control or *Mll4*$^{-/-}$ B16-Ova cells lentivirally transduced with shRNAs targeting *Gsdmd* were treated as in (d). OT-1 CD8^{+} T cell activation (e) and cytotoxic capacity (f) were analyzed by flow cytometry and shown as mean ±SEM from four or five biological replicates.

Statistical significance was determined by two-tailed unpaired t test (b, c, d, e and f). *$P < 0.05$,**$P < 0.01$, ***$P < 0.001$.

Supplementary Figure 6

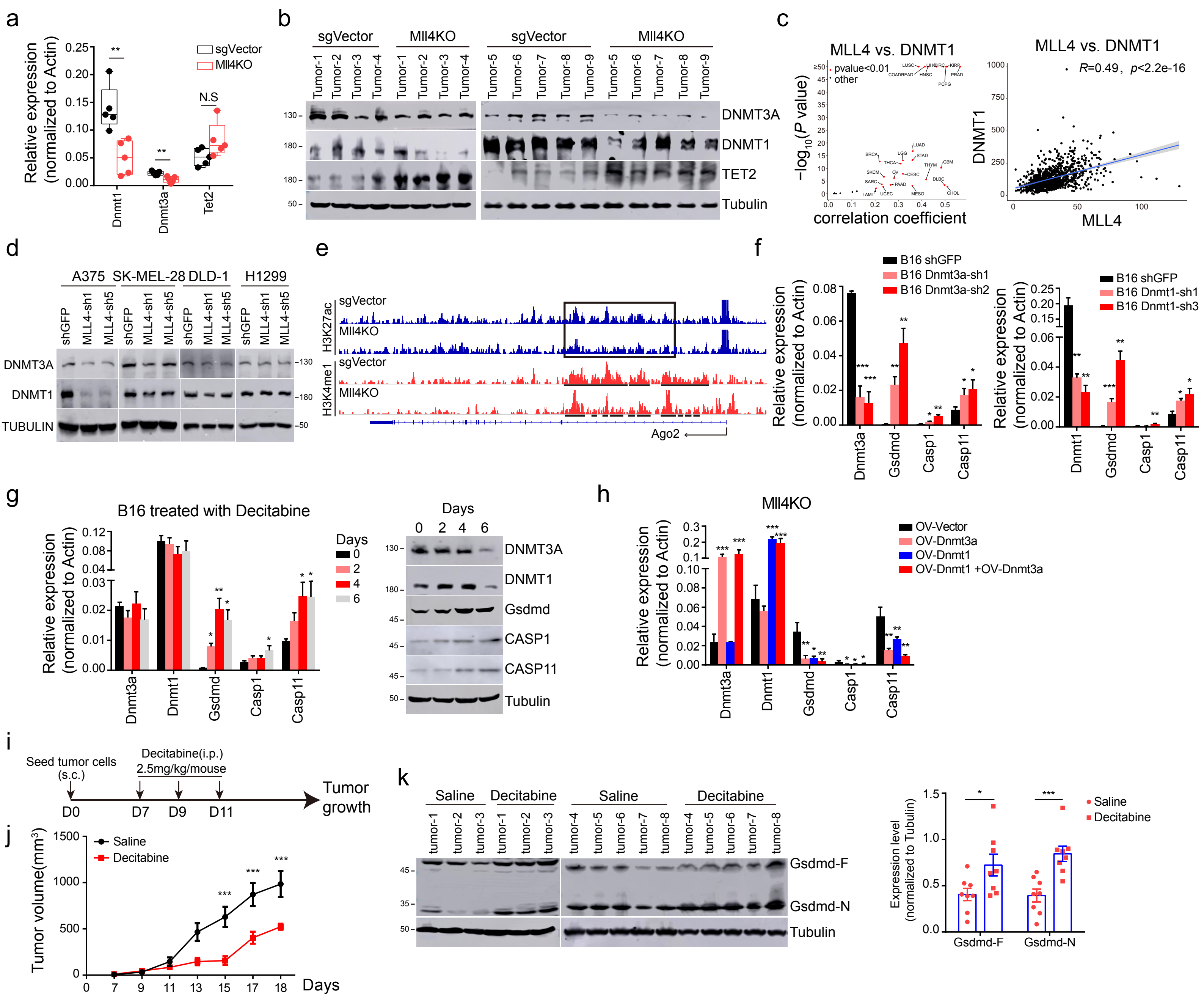

Supplementary Figure 6. MLL4 suppresses *Gsdmd* expression through promoting DNA methylation by *Dnmt3a* and *Dnmt1*, related to Figure 6.

(a). Box plot showing mRNA levels of indicated genes in control and *Mll4*$^{-/-}$ B16 tumors determined by qRT-PCR. Center line, mean; box bounds, upper and lower quartile; whisker, maximal and minimal value (n=5).

(b). Immunoblotting analyses of indicated proteins in control and *Mll4*$^{-/-}$ B16 bulk tumors.

(c). Volcano and scatter plots showing the Spearman’s correlation for the transcript levels of *MLL4* with *DNMT1* across TCGA human cancer types (left) and all CCLE cell lines (right). Blue line, linear regression fit; shaded area, 95% confidence interval.

(d). Immunoblotting analyses of indicated protein in human cancer cell lines with or without *MLL4* knockdown.

(e). IGV browser tracks showing H3K4me1 and H3K27ac ChIP-seq signal at *Ago2* locus in control and *Mll4*$^{-/-}$ B16 cells.

(f). mRNA levels of indicated gene in B16 cells depleted for *Dnmt3a* or *Dnmt1* was determined by RT-qPCR and shown as mean±SD from technical triplicates in one of biological replicates

(g). mRNA (left) and protein (right) levels of indicated gene in B16 cells treated with Decitabine for indicated time period were determined by RT-qPCR (mean ± SD, n=3) and immunoblotting, respectively.

(h). mRNA levels of indicated gene in *Mll4*$^{-/-}$ B16 cells transduced with indicated construct was determined by RT-qPCR and shown as mean±SD from technical triplicates in one of biological replicates.

(i). Schematic outline for C57BL/6J mice inoculated with B16 cells and treated with Decitabine.

(j). Tumor growth over time in C57BL/6J mice (n=8) treated as shown in (i).

(k). Immunoblotting analyses of GSDMD level in bulk tumors of C57BL/6J mice (mean ± SEM, n=8) treated with or without Decitabine.

All immunoblotts are representative from two independent experiments. Statistical significance was determined by two-tailed unpaired t (a, f, g, h and k), r cor.test (c) or two-way ANOVA (j). *P < 0.05,**P < 0.01, ***P < 0.001.

Supplementary Figure 7

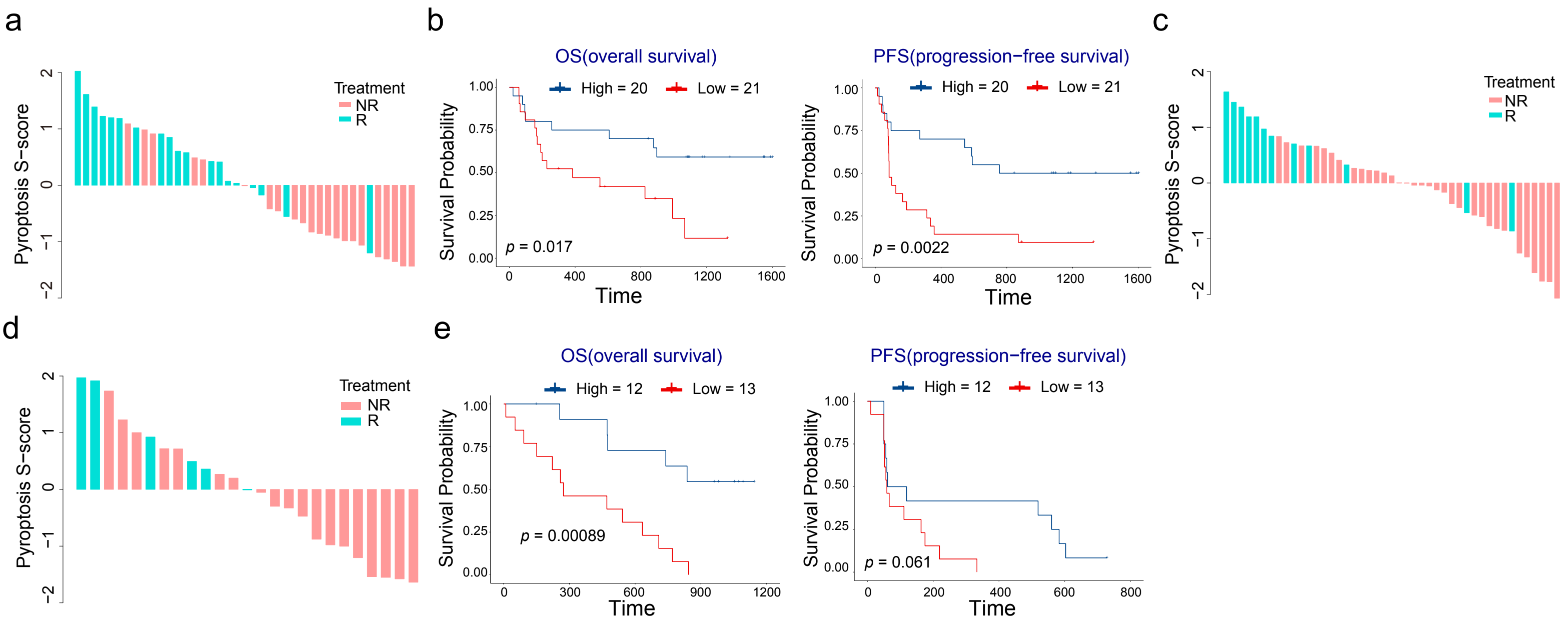

Supplementary Figure 7. A higher level of tumor cell pyroptosis correlates with a better clinical response to immune checkpoint block therapies in multiple cohorts of human cancer patients, related to Figure 7.

(a). A waterfall plot showing the pyroptosis S-score in tumors from metastatic melanoma patients who respond or do not respond to anti-PD-1 immunotherapy[3].

(b). Kaplan-Meier curves showing overall and progression-free survival of melanoma patients that were stratified by levels of pyroptosis S-score[3] (high S-score, n=20; low S-score, n=21).

(c). A waterfall plot showing the pyroptosis S-score in tumors from gastric cancer patients who respond or do not respond to anti-PD-1 immunotherapy [4].

(d). A waterfall plot showing the pyroptosis S-score in tumors from advanced melanoma patients who respond or do not respond to anti-PD-1 immunotherapy [5].

(e). Kaplan-Meier curves showing overall and progression-free survival of melanoma patients that were stratified by levels of pyroptosis S-score [5] (high S-score, n=12; low S-score, n=13).

Statistical significance was determined by log-rank test (b and e).

Supplementary Figure 8

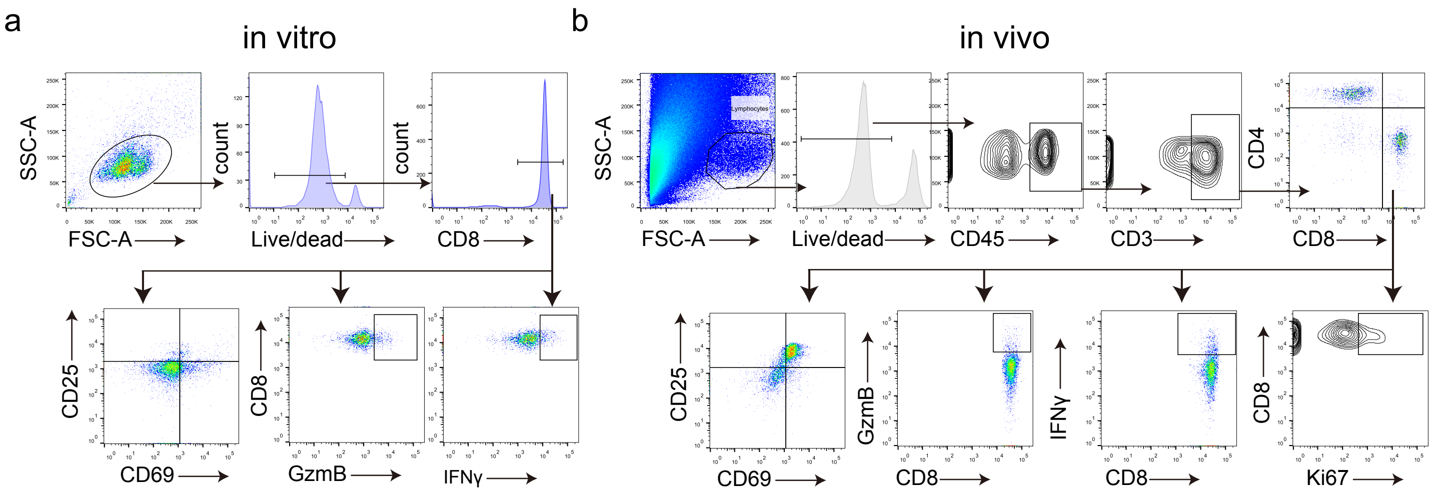

Figure S8. Gating strategies for analyzing phenotype and function of CD8^{+}T cells *in vitro* and *in vivo* by flow cytometry.

(a). Gating procedures for analyzing in vitro cultured CD8+T cells.

(b). Gating procedures for analyzing CD8+T cells in mouse tumors.

Supplementary References

1 Griffin, G. K. *et al.* Epigenetic silencing by SETDB1 suppresses tumour intrinsic immunogenicity. *Nature*, doi:10.1038/s41586-021-03520-4 (2021).

2 Li, B. *et al.* Comprehensive analyses of tumor immunity: implications for cancer immunotherapy. *Genome biology* **17**, 174, doi:10.1186/s13059-016-1028-7 (2016).

3 Gide, T. N. *et al.* Distinct Immune Cell Populations Define Response to Anti-PD-1 Monotherapy and Anti-PD-1/Anti-CTLA-4 Combined Therapy. *Cancer cell* **35**, 238-255 e236, doi:10.1016/j.ccell.2019.01.003 (2019).

4 Kim, S. T. *et al.* Comprehensive molecular characterization of clinical responses to PD-1 inhibition in metastatic gastric cancer. *Nature medicine* **24**, 1449-1458, doi:10.1038/s41591-018-0101-z (2018).

5 Riaz, N. *et al.* Tumor and Microenvironment Evolution during Immunotherapy with Nivolumab. *Cell* **171**, 934-949 e916, doi:10.1016/j.cell.2017.09.028 (2017).
