# Supplementary material for: Enhancer decommissioning by MLL4 ablation elicits dsRNA-interferon signaling and GSDMD-mediated pyroptosis to potentiate anti-tumor immunity
Source: Nat Commun. 2022 Nov 2;13:6578. doi: 10.1038/s41467-022-34253-1 (PMC9630274; doi:10.1038/s41467-022-34253-1)
Supplement: Supplementary file 3 — Description of Additional Supplementary Files [file 41467_2022_34253_MOESM3_ESM.pdf]

File Name: Supplementary Data 1

Description: Information on the DNA oligo sequence, constructs, antibodies, cell lines, software, mouse strains and other reagents.
