# Supplementary material for: Enhancer decommissioning by MLL4 ablation elicits dsRNA-interferon signaling and GSDMD-mediated pyroptosis to potentiate anti-tumor immunity
Source: Nat Commun. 2022 Nov 2;13:6578. doi: 10.1038/s41467-022-34253-1 (PMC9630274; doi:10.1038/s41467-022-34253-1)
Supplement: Supplementary file 5 — Reporting Summary [file 41467_2022_34253_MOESM5_ESM.pdf]

nature portfolio

Corresponding author(s): Deqing Hu and Xin Gao

Last updated by author(s): Oct 13, 2022

Reporting Summary

Nature Portfolio wishes to improve the reproducibility of the work that we publish. This form provides structure for consistency and transparency in reporting. For further information on Nature Portfolio policies, see our Editorial Policies and the Editorial Policy Checklist.

Statistics

For all statistical analyses, confirm that the following items are present in the figure legend, table legend, main text, or Methods section.

n/a	Confirmed	
☐	☒	The exact sample size (*n*) for each experimental group/condition, given as a discrete number and unit of measurement
☐	☒	A statement on whether measurements were taken from distinct samples or whether the same sample was measured repeatedly
☐	☒	The statistical test(s) used AND whether they are one- or two-sided<br>*Only common tests should be described solely by name; describe more complex techniques in the Methods section.*
☒	☐	A description of all covariates tested
☒	☐	A description of any assumptions or corrections, such as tests of normality and adjustment for multiple comparisons
☐	☒	A full description of the statistical parameters including central tendency (e.g. means) or other basic estimates (e.g. regression coefficient) AND variation (e.g. standard deviation) or associated estimates of uncertainty (e.g. confidence intervals)
☐	☒	For null hypothesis testing, the test statistic (e.g. *F*, *t*, *r*) with confidence intervals, effect sizes, degrees of freedom and *P* value noted<br>*Give P values as exact values whenever suitable.*
☒	☐	For Bayesian analysis, information on the choice of priors and Markov chain Monte Carlo settings
☒	☐	For hierarchical and complex designs, identification of the appropriate level for tests and full reporting of outcomes
☒	☐	Estimates of effect sizes (e.g. Cohen's *d*, Pearson's *r*), indicating how they were calculated

Our web collection on statistics for biologists contains articles on many of the points above.

Software and code

Policy information about availability of computer code

Data collection	No software was used for data collection.
Data analysis	Image J (Windows, bundles with 64-bit Java 8), Graphpad Prism 6, FlowJo, FastQC (0.11.9), Trim Galore (0.6.4), HISAT (2.2.1), GSEA, Bowtie2 (2.4.3), Samtools (1.7), Picard (2.26.5), deepTools (2.0), Integrative Genomics Viewer (2.11.1), MACS2 (2.2.7.1)

For manuscripts utilizing custom algorithms or software that are central to the research but not yet described in published literature, software must be made available to editors and reviewers. We strongly encourage code deposition in a community repository (e.g. GitHub). See the Nature Portfolio guidelines for submitting code & software for further information.

Data

Policy information about availability of data

All manuscripts must include a data availability statement. This statement should provide the following information, where applicable:

- Accession codes, unique identifiers, or web links for publicly available datasets
- A description of any restrictions on data availability
- For clinical datasets or third party data, please ensure that the statement adheres to our policy

The raw and processed ChIP-seq and RNA-seq datasets generated in this study have been deposited into Gene Expression Omnibus (GEO) under the series entry number of GSE192714 and can be downloaded from the link below: https://www.ncbi.nlm.nih.gov/geo/query/acc.cgi?acc=GSE192714. The in vitro and in vivo CRISPR/Cas9 screening data re-analyzed in this study are available as supplementary materials in Pan, et al (https://www.science.org/doi/suppl/10.1126/science.aao1710/suppl_file/aao1710_pan_sm.pdf) and Griffin, et al (https://static-content.springer.com/esm/art%3A10.1038%2Fs41586-021-03520-4/MediaObjects/41586_2021_3520_MOESM3_ESM.xlsx). The genomic sequencing datasets used for analyzing the association of MLL4 mutational status with clinical response to PD-L1-PD1 blockade therapies were available as supplementary materials in Hugo, et al (https://www.science.org/doi/suppl/10.1126/science.aad0095/

suppl_file/tables1.mutation_list_all_patients.xlsx, https://www.science.org/doi/suppl/10.1126/science.aad0095/suppl_file/tables2.clinical_and_genome_characteristics_each_patient.xlsx), Mariathasan et al (http://research-pub.gene.com/IMvigor210CoreBiologies/packageVersions/IMvigor210CoreBiologies_1.0.0.tar.gz), and Snyder, et al (https://github.com/cetienn01/Multi-Omic-aPDL1). Data used for analyzing the association of pyroptosis-related gene expression and pyroptosis S-score with therapeutic efficacy of anti-CTLA-4 and anti-PD-1 treatment in cancer patients were available as supplementary materials in Snyder, et al (https://cbioportal-datahub.s3.amazonaws.com/skcm_mskcc_2014.tar.gz), Liu, et al (https://github.com/vanallenlab/schadendorf-pd1), Gide, et al (http://tide.dfci.harvard.edu/download/release/Gide2019_PD1_Melanoma_RNASeq.tar.gz/), Kim, et al (http://tide.dfci.harvard.edu/download/release/Kim2018_PD1_Gastric_RNASeq.tar.gz)70, and Riaz, et al (https://github.com/riazn/bms038_analysis). TCGA and CCLE datasets were downloaded from https://portal.gdc.cancer.gov/ and https://sites.broadinstitute.org/ccle/datasets, respectively. Oligonucleotides sequence, antibody source and dilution, software, and additional reagents are listed in Supplementary Data 1. All remaining data associated with this study are available within the Article, Supplementary Information or Source Data file.

Field-specific reporting

Please select the one below that is the best fit for your research. If you are not sure, read the appropriate sections before making your selection.

- [x] Life sciences
- [ ] Behavioural & social sciences
- [ ] Ecological, evolutionary & environmental sciences

For a reference copy of the document with all sections, see nature.com/documents/nr-reporting-summary-flat.pdf

Life sciences study design

All studies must disclose on these points even when the disclosure is negative.

Sample size	Sample size was not predetermined by any statistic methods. Animal experiments were carried out with at least 5 mice per group to compute statistical significance, which is routinely used in literatures. For quantitative analyses of gene expression and genomic enrichment, technical triplicates from one of at least two independent experiments were usually performed to determine statistical significance, which is often employed by colleagues and are sufficient for calculating statistical significance. For Sample size and methods for statistical analysis are described in figure legends of relevant figures.
Data exclusions	We did not exclude any data for the analysis.
Replication	Results of immunoblotting, qRT-PCR, flow cytometric analysis were from at least two or three biological replicates and described in the figure legends of corresponding figures. Two biological replicates were used for generating ChIP-seq and RNA-seq datasets and the subsequent statistic analyses. At least five mice were grouped for each treatment and the exact number were described in figure legends of corresponding figures. Each experiment was performed at least twice and all replications produced similar or equivalent results or phenotypes.
Randomization	For drug and antibody treatment, same number of mice were randomly chosen into distinct treatment groups.
Blinding	Investigators were blinded to randomly allocate mice into each treatment group.

Reporting for specific materials, systems and methods

We require information from authors about some types of materials, experimental systems and methods used in many studies. Here, indicate whether each material, system or method listed is relevant to your study. If you are not sure if a list item applies to your research, read the appropriate section before selecting a response.

Materials & experimental systems

n/a	Involved in the study	
[ ]	[x]	Antibodies
[ ]	[x]	Eukaryotic cell lines
[x]	[ ]	Palaeontology and archaeology
[ ]	[x]	Animals and other organisms
[x]	[ ]	Human research participants
[x]	[ ]	Clinical data
[x]	[ ]	Dual use research of concern

Methods

n/a	Involved in the study	
[ ]	[x]	ChIP-seq
[ ]	[x]	Flow cytometry
[x]	[ ]	MRI-based neuroimaging

Antibodies

Antibodies used	The catalog numbers, suppliers and dilutions for all antibodies used in this study were described in detail in Supplementary Data 1.
Validation	Specificity for use of primary antibodies in flow cytometry, immunoblotting, ChIP-seq and immunofluorescence were either validated by shRNA- or CRISR/Cas9- mediated knockdown or knockout analyses in this study, or described in the websites of commercial suppliers or in the previous literatures that were indicated in the Supplementary Data 1.

Eukaryotic cell lines

Policy information about cell lines

Cell line source(s)	B16F10 and MC38 (a gift from Haidong Tang at Tsinghua University);HEK293T, LLC, A375, DLD-1, H1299, and SK-MEL-28 are all from ATCC. Source of the cell lines and catalog number are also described in the Supplementary Data 1.
Authentication	Cell lines were authenticated genetically by the providers and we indirectly verified their identity by their morphology, growth behavior or transcriptomic profiles.
Mycoplasma contamination	All cell lines were tested for mycoplasma contamination before their use in experiment. None of these cell lines are contaminated by mycoplasma.
Commonly misidentified lines (See ICLAC register)	No commonly misidentified lines was used.

Animals and other organisms

Policy information about studies involving animals; ARRIVE guidelines recommended for reporting animal research

Laboratory animals	6- to 8-week-old male and female C57BL/6 and BALB/c nude mice were from the Beijing Vital River Laboratory, and Rag1KO mice were purchased from GemPharmatech company. OT-I TCR transgenic mice were kindly provided by J. Yang lab (Tianjin Medical University, Tianjin, China). Mice were housed under specific pathogen free conditions in a temperature-controlled room.
Wild animals	No wild animals were used in this study.
Field-collected samples	No field-collected samples was used in this study.
Ethics oversight	Mice were used in accordance with the protocols(TMUaMEC 2020001)approved by the Institutional Animal Care and Use Committee at the Tianjin Medical University.

Note that full information on the approval of the study protocol must also be provided in the manuscript.

ChIP-seq

Data deposition

☒ Confirm that both raw and final processed data have been deposited in a public database such as GEO.

☒ Confirm that you have deposited or provided access to graph files (e.g. BED files) for the called peaks.

Data access links *May remain private before publication.*	https://www.ncbi.nlm.nih.gov/geo/query/acc.cgi?acc=GSE192714
Files in database submission	wild_type_B16_F10_cells_H3K4me1_Reads1.fq.gz<br>wild_type_B16_F10_cells_H3K4me1_Reads2.fq.gz<br>wild_type_B16_F10_cells_H3K27ac_Reads1.fq.gz<br>wild_type_B16_F10_cells_H3K27ac_Reads2.fq.gz<br>Mll4_KO_B16_F10_cells_H3K4me1_Reads1.fq.gz<br>Mll4_KO_B16_F10_cells_H3K4me1_Reads2.fq.gz<br>Mll4_KO_B16_F10_cells_H3K27ac_Reads1.fq.gz<br>Mll4_KO_B16_F10_cells_H3K27ac_Reads2.fq.gz<br>wild_type_B16_F10_cells_Input_Reads1.fq.gz<br>wild_type_B16_F10_cells_Input_Reads2.fq.gz<br>Mll4_KO_B16_F10_cells_Input_Reads1.fq.gz<br>Mll4_KO_B16_F10_cells_Input_Reads2.fq.gz<br>WT_H3K4me1.bw<br>WT_H3K27ac.bw<br>Mll4KO_H3K4me1.bw<br>Mll4KO_H3K27ac.bw<br>WT_Input.bw<br>Mll4KO_Input.bw
Genome browser session (e.g. UCSC)	https://www.igv.org/web/app/

Methodology

Replicates	Genomic sites with enrichment of a particular histone modificaiton was identified by ChIP-seq analysis and further validated by ChIP-qPCR in an independent biological replicate as shown in the relevant figures.
Sequencing depth	Sample Total_reads Uniquely_mapping_reads Length_of_reads Paired-_or_single-end

	wild_type_B16_F10_cells_H3K4me1 97216104 77315967 150bp paired<br>wild_type_B16_F10_cells_H3K27ac 91162280 12228724 150bp paired<br>Mll4_KO_B16_F10_cells_H3K4me1 96773376 67286528 150bp paired<br>Mll4_KO_B16_F10_cells_H3K27ac 87170282 11551512 150bp paired<br>wild_type_B16_F10_cells_Input 16520070 11407210 150bp paired<br>Mll4_KO_B16_F10_cells_Input 18252332 12454196 150bp paired
Antibodies	The information of antibodies have provided in supplemental Table 1.
Peak calling parameters	MACS2 (2.2.7.1) was used to call peaks at FDR cutoff of 0.05.
Data quality	The quality, adapter content and duplication rate of raw paired-end reads were confirmed by FastQC (0.11.9) with default parameters, and reads were trimmed by Trim Galore (0.6.4).<br>Sample Peak_count<br>wild_type_B16_F10_cells_H3K4me1 25897<br>wild_type_B16_F10_cells_H3K27ac 22393<br>Mll4_KO_B16_F10_cells_H3K4me1 15885<br>Mll4_KO_B16_F10_cells_H3K27ac 16459
Software	Raw paired-end reads of H3K4me1 and H3K27ac were trimmed by Trim Galore (0.6.4), and then aligned to mouse genome mm10 using Bowtie2 (2.4.3) with default parameters. Samtools (1.7) was used to remove all unmapped reads and keep one assigned position with the best matched score of non-uniquely mapped reads. Next, PCR duplicates were removed using Picard (2.26.5). For subsequent analyses, reads were extended to 200 bp and normalized to Reads Per Kilobase per Million mapped reads (RPKM) to generate bigwig files by deepTools (2.0). The bigwig track files were visualized with Integrative Genomics Viewer (2.11.1). MACS2 (2.2.7.1) was used to call peaks at FDR cutoff of 0.05. ROSE_main.py function from ROSE (http://younglab.wi.mit.edu/super_enhancer_code.html) was used to identify typical- or super-enhancers based on H3K27ac ChIP-seq signals with parameters of "-s 4000 -t 2500". ROSE_geneMapper.py function was used to annotate enhancers by their nearest genes. The expression difference of these genes between indicated groups were identified by two-sided Mann-Whitney U test. Heatmaps and average intensity curves of ChIP-seq signals for H3K27ac and H3K4me1 on typical- or super- enhancer regions were generated by deepTools (2.0).

Flow Cytometry

Plots

Confirm that:

- [x] The axis labels state the marker and fluorochrome used (e.g. CD4-FITC).
- [x] The axis scales are clearly visible. Include numbers along axes only for bottom left plot of group (a 'group' is an analysis of identical markers).
- [ ] All plots are contour plots with outliers or pseudocolor plots.
- [x] A numerical value for number of cells or percentage (with statistics) is provided.

Methodology

Sample preparation	Methods for the preparation of cells and their source were described in figure legends and Materials and Method section.
Instrument	FACS Celesta
Software	FlowJo
Cell population abundance	Abundance of sorted cells were validated by overlapping them into pre-sorted cells using Flowjo software.
Gating strategy	All gates were set based on FMO (full-minus one) stains and isotype control antibodies after appropriate compensation using single-stained compensation controls.

- [x] Tick this box to confirm that a figure exemplifying the gating strategy is provided in the Supplementary Information.
